# Economic evaluation of a rehabilitation program integrating exercise, self-management, and active coping strategies for chronic knee pain

**DOI:** 10.1002/art.23011

**Published:** 2007-10-15

**Authors:** M V Hurley, N E Walsh, H L Mitchell, T J Pimm, E Williamson, R H Jones, B C Reeves, P A Dieppe, A Patel

**Affiliations:** 1 King's College LondonLondon, UK; 2 University of the West of EnglandBristol, UK; 3 DeMontfort UniversityLeicester, UK; 4 Buckinghamshire Hospitals National Health Service TrustAylesbury, UK; 5 London School of Hygiene and Tropical MedicineLondon, UK; 6 University of BristolBristol, UK; 7 MRC Health Services Research Collaboration, University of BristolBristol, UK

**Keywords:** Economic evaluation, Rehabilitation, Knee pain

## Abstract

**Objective:**

To conduct an economic evaluation of the Enabling Self-Management and Coping with Arthritic Knee Pain through Exercise (ESCAPE-knee pain) program.

**Methods:**

Alongside a clinical trial, we estimated the costs of usual primary care and participation in ESCAPE-knee pain delivered to individuals (Indiv-rehab) or groups of 8 participants (Grp-rehab). Information on resource use and informal care received was collected during face-to-face interviews. Cost-effectiveness and cost-utility were assessed from between-group differences in costs, function (primary clinical outcome), and quality-adjusted life years (QALYs). Cost-effectiveness acceptability curves were constructed to represent uncertainty around cost-effectiveness.

**Results:**

Rehabilitation (regardless of whether Indiv-rehab or Grp-rehab) cost £224 (95% confidence interval [95% CI] £184, £262) more per person than usual primary care. The probability of rehabilitation being more cost-effective than usual primary care was 90% if decision makers were willing to pay £1,900 for improvements in functioning. Indiv-rehab cost £314/person and Grp-rehab £125/person. Indiv-rehab cost £189 (95% CI £168, £208) more per person than Grp-rehab. The probability of Indiv-rehab being more cost-effective than Grp-rehab increased as willingness to pay (WTP) increased, reaching 50% probability at WTP £5,500. The lack of differences in QALYs across the arms led to lower probabilities of cost-effectiveness based on this outcome.

**Conclusion:**

Provision of ESCAPE-knee pain had small cost implications, but it was more likely to be cost-effective in improving function than usual primary care. Group rehabilitation reduces costs without compromising clinical effectiveness, increasing probability of cost-effectiveness.

## INTRODUCTION

Chronic knee pain is a major cause of disability (1–6). Health and social care expenditure (3,7–9) on this condition will increase as more people live longer (10), and may exceed predictions based on actual information as patterns of consultation change (11) and obesity and sedentary lifestyles increase (12,13).

Knee pain is usually managed with analgesia, despite concerns about safety (9,14–16), efficacy, and direct and indirect costs of medication (17). Functional impairment resulting from knee pain is frequently overlooked despite the disability this causes and its role in the etiology of chronic ill health, e.g., diabetes, cardiovascular disease, obesity, depression (18). Functioning can be improved through self-management interventions (19,20) and exercise (21–23). However, complex, expensive rehabilitation programs benefit few individuals. Effective and affordable interventions are needed that can be administered to large numbers of people.

We have demonstrated that a rehabilitation program integrating exercise, patient education, and self-management strategies (Enabling Self-Management and Coping with Arthritic Knee Pain Through Exercise [ESCAPE-knee pain]) improves functioning (24,25). However, when planning health care for local communities, commissioners also need cost and cost-effectiveness information. This report details the economic evaluation of ESCAPE-knee pain.

## PATIENTS AND METHODS

### Design, setting, and participants

A pragmatic, cluster randomized controlled trial was carried out and analyzed in accordance with the prespecified protocol. Inclusion and exclusion criteria, randomization, and clinical outcomes are described elsewhere (25). Briefly, participants had to be age 50 years or older and have attended their primary care practice reporting mild, moderate, or severe knee pain of more than 6 months' duration. Exclusion criteria were as follows: lower limb arthroplasty, physiotherapy for knee pain during the preceding 12 months, intraarticular injections during the preceding 6 months, unstable medical conditions, inability/unwillingness to exercise, wheelchair bound, and inability to understand English. The study was approved by the local ethics committee and participants gave informed consent.

### Interventions

The units of randomization were primary care practices, which were allocated to 1) usual primary care, 2) usual primary care plus the rehabilitation program administered to individual participants (Indiv-rehab), or 3) usual primary care plus the rehabilitation program delivered to groups of 8 participants (Grp-rehab). The content and format of the rehabilitation program were similar for Indiv-rehab and Grp-rehab, and consisted of 12 supervised sessions (twice weekly for 6 weeks). For 10–15 minutes of each session, the supervising physiotherapist facilitated a discussion on a specific topic, advising and suggesting simple coping strategies. Then for 30–45 minutes, participants performed simple exercises to improve functioning; these exercises were personalized and progressed according to each participant's ability. (For details of the program see www.kcl.ac.uk/gppc/escape.) We hypothesized that rehabilitation (disregarding whether costs were incurred during Indiv-rehab or Grp-rehab) would cost more than usual primary care, and that Indiv-rehab would cost more than Grp-rehab.

### Outcomes

The a priori primary outcome was a clinically meaningful improvement (15% from baseline level) in self-reported functioning, assessed using the physical function subscore of the Western Ontario and McMaster Universities Osteoarthritis Index (WOMAC-func) (26), 6 months after completion of rehabilitation (7.5 months after baseline assessment when 6-week intervention or usual primary care control period was included). Quality of life was assessed using the EuroQoL (EQ-5D) (27). Quality-adjusted life years (QALYs) were calculated by applying UK general population utility weights (28) to the 5-dimensional health state descriptions for each participant.

### Resource use

Management of all participants' knee problems continued at the primary care physician's discretion, and was documented at assessment. Data on resource use were collected retrospectively at baseline and followup using the Client Services Receipt Inventory (CSRI) (29), which was customized to ensure it was relevant to individuals with knee pain. The CSRI was administered as a face-to-face clinical interview at the baseline assessment covering the 6 months prior to baseline assessment, and at the followup assessment covering the period between baseline assessment and 6 months after completion of the rehabilitation program (7.5 months from baseline). Data collected included use of health and voluntary resources, use of rehabilitation facilities, patient costs (e.g., travel, medication, over-the-counter drugs), time off work, social security benefits, and informal care. The resources used during the Indiv-rehab and Grp-rehab programs were recorded separately to estimate the costs of rehabilitation (disregarding whether costs were incurred during Indiv-rehab or Grp-rehab).

### Unit costs

Costs were calculated for each participant by multiplying resource use data by unit costs based on national statistics (Appendix A, available at the *Arthritis Care & Research* Web site at http://www.interscience.wiley.com/jpages/0004-3591:1/suppmat/index.html). Informal care costs were estimated using the replacement cost approach by applying the unit cost of a social services home help worker.

Unit costs of rehabilitation were estimated from a health care perspective. Because resource inputs for a session of Indiv-rehab and Grp-rehab did not vary between sessions or participants, unit costs for each intervention were estimated as an average cost per person per session. Intervention unit cost estimations (described below) were then applied to individual-level data, according to the number of sessions attended, to derive a total intervention cost for each participant. Calculation of these unit costs involved first identifying all resource inputs normally associated with running one session of each program (e.g., contact and noncontact time with the therapist, capital costs, overhead costs, exercise equipment, materials/photocopying). Second, the cost of each of these components was estimated. A research associate (hourly rate £18.78 [$30]) supervised all rehabilitation sessions. Average face-to-face contact time was 45 minutes for each Indiv-rehab session and 60 minutes for each Grp-rehab session; these times were adjusted for group size, then multiplied by the hourly cost to obtain per-person face-to-face contact time costs of £14.09 ($23) and £2.35 ($4) for Indiv-rehab and Grp-rehab sessions, respectively. Average noncontact time (prepare session, write up notes) associated with each person for each session was 30 minutes for Indiv-rehab and Grp-rehab. This was multiplied by the hourly cost to obtain per-person non–face-to-face contact time costs of £9.39 ($15). Third, the costs of all resource components were summed to obtain a total cost per session for each intervention. Finally, the total cost for Grp-rehab was divided by the average group size (n = 8) to estimate its cost per person per session.

All costs were standardized to 2003/2004 prices, using the UK National Health Service Executive hospital and community health services or personal social services inflation indices (30). Discounting costs was unnecessary because of the short assessment period (6 months). Costs are presented in British pounds sterling (£) and converted to US dollars ($) based on a 2003 purchasing power parity (conversion rate: £1 = $1.613) that equalizes the purchasing power of the 2 currencies (31).

### Statistical analyses

Because the CSRI was administered by interview, missing data were minimal. However, if use of a particular service was reported but the quantity was not specified, within-group mean costs for that item for participants who did have data at the same assessment point were used to impute missing quantities. Where information about the use of a particular service was missing, we assumed that service had not been used and a zero cost was applied. For missing EQ-5D values at 6-month followup, values were imputed using the last value carried forward. Values could not be imputed for missing EQ-5D scores at baseline.

QALYs were calculated as gains since baseline. This calculation incorporated values from a 6-week measurement. Six-week and 6-month health state measurements were assumed to represent the time since the last assessment. Proportional QALY gains for these 2 time points were summed to represent a total 6-month gain since baseline.

Intent-to-treat analyses were conducted. Mean costs per group were based on participant-level costs, unadjusted for clustering. However, a cluster adjustment was included in the examination of differences in cost between groups. Differences were compared using linear regression with the cluster adjustment procedure in Stata v8.2 (StataCorp, College Station, TX). Each cost category was adjusted for the baseline value of the same category. To allow for the non-normal distribution commonly associated with cost data, 1,000 nonparametric bootstrap replications were performed to obtain estimates of mean differences and 95% confidence intervals (95% CIs). The primary perspective was that of health and social care, the secondary perspective that of society. Analyses were performed using SPSS 12.0.1 (SPSS, Chicago, IL) and Stata 8.2 (StataCorp).

### Cost-effectiveness analyses

The main cost-effectiveness analyses focused on between-group differences in total costs and WOMAC-func (the primary outcome) at 6-month followup. In addition, a cost-utility analysis was conducted by combining cost and QALY data.

Uncertainty concerning cost-effectiveness results was represented by cost-effectiveness acceptability curves (CEACs) using the net benefit approach (32,33). CEACs show the probability that one intervention is cost–effective compared with alternative(s), given a range of willingness-to-pay values (WTP) that a health care commissioner may be prepared to accept for improvements in outcome.

For CEACs based on WOMAC-func, we compared Indiv-rehab with Grp-rehab, and rehabilitation with usual care; these comparisons were made from both the health and social care perspective and the societal perspective. To make the cost-effectiveness analysis meaningful to health care commissioners, WOMAC-func was examined as the proportion of participants showing clinically meaningful improvements, i.e., at least 15% from baseline (34). A total of 1,000 bootstrap samples of costs and effects data were used to generate distributions of the mean costs and effects for each arm to deal with the dichotomous nature of the outcome measure.

For the CEACs based on QALYs, each intervention was compared with the alternatives simultaneously, and analyses were only conducted from a health and social care perspective in line with the current decision-making framework used by the UK National Institute for Health and Clinical Excellence. CEACs based on QALYs were based on observed data. Both sets of CEACs were constructed using Excel (Microsoft, Redmond, WA) program macros created by Elizabeth Fenwick (University of York, UK).

### Sensitivity analyses

Two sets of sensitivity analyses were performed. First, we tested the effect of alternative assumptions for the calculation of unit costs of rehabilitation. Second, we tested the impact of alternative assumptions for the calculation of total health/social care costs and total societal costs. We examined rehabilitation unit costs under 6 alternative assumptions: supervision by a junior therapist; reducing groups to 6 participants; increasing groups to 10 participants; widening the cost difference between the interventions by 50%; narrowing the cost difference by 50%; taking a societal perspective that includes participant's travel expenses, travel time to and from, and participation in rehabilitation sessions.

For the examination of total costs, sensitivity analyses were performed to examine alternative assumptions concerning medications and informal care. There were no data on the duration of medication use, so we assumed that all reported medications were taken for the full period of assessment. Because this may have overestimated medication costs, costs were reestimated assuming medications were taken for half the assessment period (i.e., medication costs reduced by 50%). The impact of this was tested in the economic evaluation from the health/social care perspective (sensitivity analysis 1) and from the societal perspective (sensitivity analysis 2). Participants also had difficulty estimating the duration of informal care inputs and, specifically, identifying the inputs necessitated by having knee pain, so we excluded these from total societal costs (sensitivity analysis 3).

## RESULTS

### Participant characteristics

A total of 418 individuals (294 women) were recruited. Baseline anthropometric, clinical, and sociodemographic characteristics were balanced across trial arms; baseline mean age was 66 years (range 50–91 years), mean height 1.64 meters (range 1.30–1.97), body mass 81 kg (range 47–139), body mass index 30 kg/m^2^ (range 18–51), WOMAC-func 27.2 points (95% CI 25.7, 28.6), and median duration of symptoms was 6 years (interquartile range 3–12). The majority of participants received some state benefits, predominantly a pension. There were some between-group imbalances in receipt of social security benefits at baseline (usual care: 118 [84%] of 140; Indiv-rehab: 108 [74%] of 146; Grp-rehab: 120 [91%] of 132). At 6-month followup, economic data were available for 338 (81%) participants and completion rates were balanced between trial arms (usual care: 110 [79%] of 140; Indiv-rehab: 121 [83%] of 146; Grp-rehab: 107 [81%] of 132).

From the health/social care (although not societal) perspective, participants in the usual care arm who remained in the study at the 6-month assessment had lower total baseline costs and higher (better) baseline utility values than participants who withdrew. Because these differences were not present in the intervention arms, the data of usual care participants remaining in the study were biased toward participants with better quality of life and/or lower costs, causing the cost-effectiveness of rehabilitation to be underestimated.

### Outcome results

At baseline there were no differences between the groups in costs or utilities (Table 1). There were some changes in costs and QALYs at the 6-month assessment, but no overall differences between any of the trial arms (Table 2). WOMAC-func improved in participants in Indiv-rehab and Grp-rehab, and the proportion of participants with clinically meaningful improvement in functioning was greater following rehabilitation than usual primary care (121 of 226 versus 47 of 113, respectively; χ^2^ = 4.301, *P* = 0.038) (Table 2)

**Table 1:** Mean and mean differences in costs (£, 2003/2004) and quality-adjusted life years at baseline*

	Usual care†	Rehabilitation†	Indiv-rehab†	Grp-rehab†	Rehabilitation vs usual care, mean difference (95% CI)‡	Indiv-rehab vs Grp-rehab, mean difference (95% CI)‡
Health and social care						
Secondary care	53.05 ± 170	43.15 ± 132	46.33 ± 132	39.64 ± 132	−10 (−53, 24)	7(−20, 39)
Community-based care	29.81 ± 46	32.55 ± 61	34.86 ± 73	30.00 ± 44	3 (−8, 13)	5 (−10, 21)
Medication	19.90 ± 37	22.44 ± 39	22.17 ± 38	22.74 ± 41	3 (−7, 11)	−1 (−11, 11)
Total	102.77 ± 185	98.15 ± 152	103.36 ± 159	92.38 ± 144	−5 (−51, 30)	11 (−23, 52)
Patient/family						
Health/social care services	5.20 ± 32	6.71 ± 44	3.46 ± 28	10.30 ± 57	2 (−5, 8)	−7 (−19, 2)
Medication	34.44 ± 55	26.65 ± 43	25.37 ± 41	28.06 ± 46	−8 (−16, 1)	−3 (−14, 7)
Informal care	349.43 ± 574	317.76 ± 569	310.16 ± 488	326.17 ± 648	−32 (−154, 80)	−16 (−174, 134)
Other expenses	132.03 ± 342	79.33 ± 236	79.84 ± 224	78.76 ± 250	−53 (−116, 0)	1 (−60, 68)
Total	521.09 ± 794	430.45 ± 678	418.83 ± 563	443.30 ± 788	−91 (−249, 50)	−24 (−185, 147)
Other						
Time off work by patient	199.85 ± 1,575	148.62 ± 1,243	187.50 ± 1,516	105.62 ± 850	−51 (−323, 187)	82 (−163, 309)
Time off work by carer	0.19 ± 1	0.34 ± 3	0.59 ± 4	0.07 ± 0.78	0 (0, 0)	1 (0, 1)
Social security benefits	2,896.09 ± 2,897	2,643.20 ± 2,422	2,359.82 ± 2,399	2,956.63 ± 2,417	−253 (−999, 462)	−597 (−1,350, 160)
Total	3,096.13 ± 3,259	2,792.16 ± 2,620	2,547.91 ± 2,761	3,062.31 ± 2,438	−304 (−1,060, 406)	−514 (−1,279, 277)
Total costs						
Health/social care perspective	102.77 ± 185	98.15 ± 152	103.36 ± 159	92.38 ± 144	−5 (−51, 30)	11 (−23, 52)
Societal perspective	3,719.99 ± 3,514	3,320.75 ± 2,881	3,070.10 ± 2,925	3,597.99 ± 2,817	−399 (−1,180, 364)	−528 (−1,372, 362)
Sensitivity analysis 1	92.90 ± 180	86.92 ± 148	92.27 ± 154	81.01 ± 141	−6 (−52, 28)	11 (−23, 50)
Sensitivity analysis 2	3,709.31 ± 3,511	3,309.28 ± 2,882	3,058.85 ± 2,926	3,586.28 ± 2,818	−400 (−1,182, 365)	−527 (−1,372, 363)
Sensitivity analysis 3	3,370.56 ± 3,366	3,002.99 ± 2,706	2,759.94 ± 2,806	3,271.82 ± 2,574	−367 (−1,136, 344)	−512 (−1,317, 313)
Outcomes						
Utilities	0.60 ± 0.30	0.60 ± 0.28	0.60 ± 0.29	0.60 ± 0.28	0 (−0.1, 0.1)	0 (−0.1, 0.1)

* Values are the mean ± SD unless otherwise indicated. Conversion rate to US dollars at 2003 purchasing power parity: £1 = $1.613. £ = English pounds sterling; rehabilitation = costs of individual and group rehabilitation programs combined; Indiv-rehab = rehabilitation program delivered to individual participants; Grp-rehab = rehabilitation program delivered to groups of 8 participants; 95% CI = 95% confidence interval; sensitivity analysis 1 = health service perspective with lower medication unit costs; sensitivity analysis 2 = societal perspective with lower medication unit costs; sensitivity analysis 3 = societal perspective excluding informal care costs.

† Mean costs per group are based on individual-level means, unadjusted for clusters.

‡ Cluster-adjusted mean differences and confidence intervals, obtained from 1,000 bootstrap replications.

**Table 2:** Mean and mean differences in costs (£, 2003/2004) and QALYs, and differences in proportion of individuals improving on WOMAC function score at 6 months*

					Rehabilitation vs usual care, mean difference (95% CI)‡		Indiv-rehab vs Grp-rehab, mean difference (95% CI)‡	
						
	Usual care	Rehabilitation	Indiv-rehab†	Grp-rehab†	χ^2^(*P* value)	Mean difference (95% cl)	χ^2^(*P* value)	Mean difference (95% cl)
Knee rehabilitation								
Health care perspective	0.00 ± 0	224.26 ± 131	314.26 ± 111	125.41 ± 61		224 (184, 262)§		189 (168, 208)§
Societal perspective	0.00 ± 0	358.68 ± 177	455.20 ± 161	252.65 ± 125		359 (313, 400)§		203 (168, 235)§
Health and social care								
Secondary care	133.87 ± 778	60.42 ± 372	24.97 ± 173	100.50 ± 509		−75 (−255, 50)		−76 (−210, 17)
Community-based care	16.87 ± 48	17.97 ± 72	24.82 ± 90	10.23 ± 42		−2 (−15, 10)		14 (−6, 33)
Medication	12.90 ± 24	15.01 ± 32	11.79 ± 28	18.65 ± 35		−1 (−7, 5)		−6 (−11, 1)
Total	163.63 ± 809	93.40 ± 392	61.58 ± 196	129.38 ± 533		−82 (−283, 52)		−68 (−217, 28)
Patient/family								
Health/social care services	3.30 ± 18	4.31 ± 28	3.37 ± 16.78	5.38 ± 37		1 (−2, 4)		0 (−3, 4)
Medication	31.82 ± 51	25.55 ± 42	27.65 ± 47	23.18 ± 35		−4 (−12, 3)		5 (−3, 13)
Informal care	287.49 ± 474	259.79 ± 421	264.71 ± 386	254.23 ± 460		−45 (−145, 48)		33 (−33, 103)
Other expenses	118.01 ± 245	77.74 ± 222	76.28 ± 186	79.39 ± 257		−19 (−54, 15)		−3 (−34, 36)
Total	411.19 ± 616	328.15 ± 496	343.06 ± 438	311.54 ± 554		−75 (−206, 42)		54 (−16, 130)
Other								
Time off work by patient	14.19 ± 117	125.24 ± 1,197	112.51 ± 1,158	139.63 ± 1,245		180 (−11, 368)		−91 (−185, 14)
Time off work by carer	0.41 ± 4	0.02 ± 0.30	0.00 ± 0	0.04 ± 0.44		0 (−1, 1)		0 (0, 0)
Social security benefits	2,213.10 ± 2,328	2,131.28 ± 1,997	1,838.56 ± 1,939	2,462.31 ± 2,018		108 (−87, 330)		−20 (−186, 157)
Total	2,227.70 ± 2,324	2,256.54 ± 2,361	1,951.07 ± 2,224	2,601.98 ± 2,473		324 (30, 571)§		−27 (−326, 227)
Total costs								
Health/social care perspective	163.63 ± 809	344.95 ± 394	408.47 ± 212	273.13 ± 522		169 (−35, 302)		135 (−12, 230)
Societal perspective	2,831.95 ± 2,693	3,120.08 ± 2,547	2,887.23 ± 2,319	3,383.39 ± 2,769		584 (129, 927)§		149 (−165, 407)
Sensitivity analysis 1	157.24 ± 808	337.48 ± 394	402.58 ± 210	263.86 ± 521		172 (−28, 303)		138 (−11, 234)
Sensitivity analysis 2	2,824.98 ± 2,689	3,112.27 ± 2,546	2,881.05 ± 2,317	3,373.74 ± 2,769		585 (132, 926)§		151 (−162, 409)
Sensitivity analysis 3	2,505.66 ± 2,579	2,830.02 ± 2,437	2,610.85 ± 2,278	3,074.00 ± 2,592		623 (205, 943)§		151 (−176, 442)
Outcomes								
QALY gains	0.0096 ± 0.07	0.0009 ± 0.07	−0.0034 ± 0.07	0.0057 ± 0.08		−0.009 (−0.03, 0.01)		0.009 (−0.03, 0.106)
WOMAC-func, no./total no. (%)¶	47/113 (42)	121/226 (54)	65/118 (55)	56/108 (52)	4.301 (0.038)§		0.237 (0.626)	

* Values are the mean ± SD unless otherwise indicated. Conversion rate to US dollars at 2003 center power parity: £1 = $1.613. QALYs = quality-adjusted life years; WOMAC-func = physical function subscore of the Western Ontario and McMaster Universities Osteoarthritis Index; see Table 1 for additional definitions.

† Mean values per group are based on individual-level means, unadjusted for clusters.

‡ Cluster-adjusted mean differences and confidence intervals, obtained from 1,000 bootstrap replications. Costs were adjusted for baseline values of the same variable.

§ Significant difference between compared groups.

¶ Number of individuals who improved on WOMAC-func by at least 15% from baseline.

### Used resources

Resource use was low, with outpatient and general practitioner visits the most commonly used services. Mean visits among users of general practitioner services were 2.6 for usual care, 1.7 for Indiv-rehab, and 1.9 for Grp-rehab. Mean visits among users of outpatient services were 2.3 for usual care, 1.3 for Indiv-rehab, and 2.2 for Grp-rehab. Although approximately two-thirds of participants received some level of informal care, it was at relatively low levels (Table 3).

**Table 3:** Number of participants and average weekly duration of informal care inputs at 6-month followup*

	Usual primary care	Indiv-rehab	Grp-rehab
Personal care	0.12 ± 0.5	0.21 ± 1.1	0.16 ± 1.4
Home maintenance	0.20 ± 0.5	0.24 ± 0.5	0.26 ± 0.5
Housework/laundry	0.94 ± 1.9	0.84 ± 1.7	0.68 ± 1.9
Providing transport	0.25 ± 0.7	0.23 ± 0.7	0.36 ± 1.0
Preparing meals	0.44 ± 2.3	0.26 ± 1.2	0.23 ± 1.4
Gardening	0.44 ± 0.9	0.38 ± 0.8	0.34 ± 0.7
Shopping	0.80 ± 1.2	0.77 ± 1.0	0.65 ± 0.9
Total hours per week	3.2 ± 5.2	2.9 ± 4.3	2.8 ± 5.1
Number of participants who received any care (%)	65 (59)	66 (55)	67 (63)

* Values are the mean ± SD unless otherwise indicated. See Table 1 for definitions.

### Rehabilitation costs

#### Mean costs

Usual care incurred no rehabilitation costs. Indiv-rehab cost £30.40 ($49) per session per person (Table 4). Accounting for attendance rates, mean costs for Indiv-rehab were £314 ($507) per person (Tables 2 and Tables 4). Grp-rehab cost £14.33 ($23) per session per person (a total cost of £114.64 [$185] per session based on an average group size of 8 participants) (Table 4). Mean cost of Grp-rehab was £125 ($202) per person (Tables 2 and 4)

**Table 4:** Unit costs of intervention, mean ± SD intervention costs, and mean differences in costs (95% CI)*

			Indiv-rehab		Grp-rehab		
					
Scenario	Rehabilitation, mean ± SD cost†	Rehabilitation vs care mean difference(95%CI)‡	Unit cost§	Mean ±SD cost†	Unit cost§	Mean ±SD cost†	Indiv-rehab vs Grp-rehab, mean difference (95% CI)‡
Base case	224 ± 131	224 (184, 262)	30.40	314 ± 111	14.33	125 ± 61	189 (168, 208)
Junior supervisor	171 ± 101	171 (140, 201)	23.21	241 ± 85	10.74	95 ± 46	146 (130, 160)
Smaller groups	229 ± 129	229 (190, 265)	30.40	314 ± 111	15.39	134 ± 66	180 (159, 199)
Larger groups	222 ± 133	222 (181, 261)	30.40	314 ± 111	13.69	120 ± 59	194 (174, 212)
Wider cost difference	306 ± 214	306 (233, 376)	45.60	470 ± 166	14.33	125 ± 61	344 (316, 369)
Narrower cost differences	253 ± 120	253 (224, 280)	30.40	314 ± 111	21.50	187 ± 92	128 (103, 151)
Societal perspective	359 ± 177	359 (313, 400)	44.19	455 ± 161	29.26	253 ± 125	203 (168, 235)

Usual primary care had no rehabilitation costs. Conversion rate to US dollars at 2003 purchasing power parity: £1 = $1.613. A fixed total average cost of £3.70 per-person for materials is excluded from the unit cost estimates but was included in calculation of individual-level costs. See Table 1 for definitions.

Mean costs per group are based on individual-level means, unadjusted for clusters.

Cluster-adjusted mean differences and confidence intervals, obtained from 1,000 bootstrap replications.

Unit cost per session per person.

#### Differences in mean costs

Participation in rehabilitation cost £224 (95% CI £184, £262 [$361; 95% CI $297, $423]) more than usual care, with differences of £171–£359 ($276–$579) in the alternative scenarios (Tables 2 and 4). Indiv-rehab cost £189 (95% CI £168, £208 [$305; 95% CI $271, $336]) more per person than Grp-rehab, and was more expensive than Grp-rehab under all alternative unit cost assumptions with differences of £128–£344 ($207–$555) (Tables 2 and 4).

### Total costs

There were no differences in total costs between Indiv-rehab and Grp-rehab (Table 2). Although rehabilitation did not have significantly greater costs than usual care from a health/social care perspective, it cost £584 more from a societal perspective and based on 2 sensitivity analysis scenarios (Table 2).

### Cost-effectiveness

Only participants with both cost and relevant outcome data were included in the cost-effectiveness analyses. For the WOMAC-based analyses from both perspectives, the sample sizes were as follows: 110 (79%) of 140 in usual care, 118 (81%) of 146 in Indiv-rehab, and 107 (81%) of 132 in Grp-rehab. For the QALY-based analyses, the sample sizes were 109 (78%) of 140 in usual care, 121 (83%) of 146 in Indiv-rehab, and 106 (80%) of 132 in Grp-rehab.

**Figure 1:**
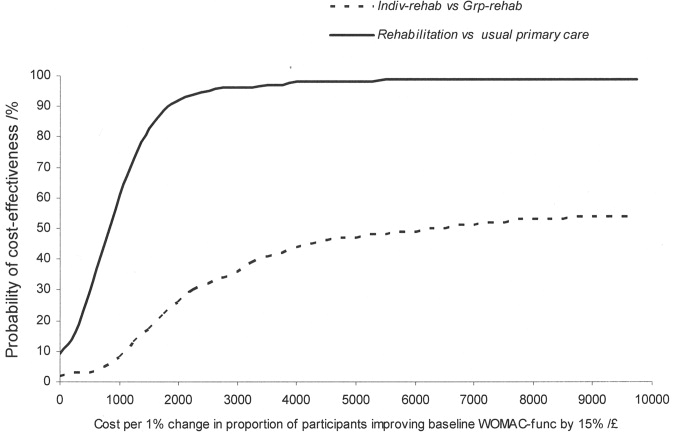
Cost-effectiveness acceptability curves: probability (given as percentage) that 1) rehabilitation (individual or group) is cost-effective compared with usual primary care and 2) Indiv-rehab is cost-effective compared with Grp-rehab, for a range of values of health care commissioners' willingness to pay for an increase in the proportion of participants improving in Western Ontario and McMaster Universities Osteoarthritis Index (WOMAC-func) by 15% at 6 months, from a health/social care perspective. Rehabilitation = costs of individual and group rehabilitation programs combined; Indiv-rehab = rehabilitation program delivered to individual participants; Grp-rehab = rehabilitation program delivered to groups of 8 participants; £ = English pounds sterling. Conversion rate to US dollars at 2003 purchasing power parity: £1 = $1.613.

If decision makers were unwilling to pay anything for an increased proportion of individuals with improved functioning, the probability that rehabilitation would be cost-effective compared with usual care was 9% (Figure 1). If health care commissioners were willing to pay ∼£800 ($1,291) for rehabilitation, there was 50% probability that supplementing usual primary care with rehabilitation was more cost-effective than usual primary care alone. This increased to >90% at WTP values ≥£1,900 (≥$3,065) (Figure 1)

Compared with Grp-rehab, Indiv-rehab had higher costs but only marginally better outcomes. If decision makers were unwilling to pay anything for an increase in the proportion of individuals improving their functioning, there was almost zero probability Indiv-rehab would be more cost-effective than Grp-rehab (Figure 1). The probability of Indiv-rehab being more cost-effective than Grp-rehab increased as commissioners' WTP increased, reaching 50% probability at WTP values >£6,000 (>$9,678).

**Figure 2:**
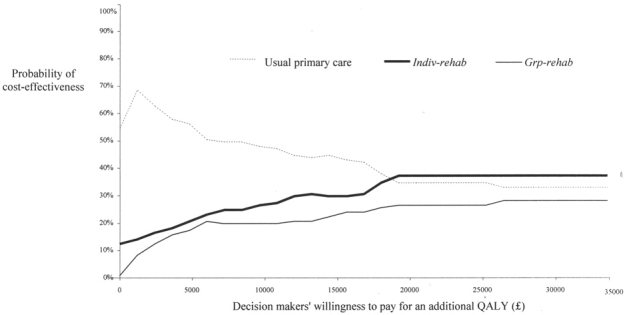
Cost-effectiveness acceptability curves: probability that each treatment strategy is cost-effective compared with the other 2, for a range of values of decision makers' willingness to pay for an additional quality-adjusted life year (QALY), from a health/social care perspective at 6 months. Indiv-rehab = rehabilitation program delivered to individual participants; Grp-rehab = rehabilitation program delivered to groups of 8 participants; £ = English pounds sterling. Conversion rate to US dollars at 2003 purchasing power parity: £1 = $1.613.

Because there was very little difference in QALYs across the arms (Table 2), CEACs based on this outcome measure showed relatively low probabilities of cost-effectiveness for both Indiv-rehab and Grp-rehab (Figure 2). It was not until threshold values were ≥£19,200 that either Indiv-rehab or Grp-rehab showed higher probabilities of cost-effectiveness than usual care. At this threshold and above, the probability that Indiv-rehab would be cost-effective compared with Grp-rehab and usual care exceeded probabilities of cost-effectiveness for the other 2 treatment strategies. However, the probability did not exceed 38% in the range of thresholds examined.

## DISCUSSION

This is the first study evaluating the costs and cost-effectiveness of an integrated exercise and self-management rehabilitation program designed to improve functioning in persons with chronic knee pain. Rehabilitation had cost implications, but at modest levels of investment was more likely to be cost-effective than usual primary care: investing £1,900 (or more) provided a 90% (or greater) chance of rehabilitation being more cost-effective than usual primary care. Administering ESCAPE-knee pain to small groups of individuals reduced its costs without compromising clinical effectiveness, increasing the probability of cost-effectiveness.

The strengths of this trial and intervention are its rigour, clinical relevance, and generalizability (35). Performing prospective interview-based economic evaluation alongside the clinical trial minimized missing data. Constructing CEACs is more informative than incremental cost-effectiveness ratios because they indicate the uncertainty associated with the findings in the absence of knowledge of how much health care commissioners would be willing to pay for meaningful improvements in functioning.

Inaccurate or biased recall due to memory and cognitive impairments is a concern in elderly individuals and may have affected the results. However, none of our participants had overt memory problems, and all were light users of health care services, had a slowly progressive condition, and lived independently, therefore we have no reason to believe that short-term recall of resource utilization was very inaccurate or biased toward over-or underestimation of resource use. Like many population-based economic evaluations with a broad perspective, it was not possible to validate self-reported resource use against all relevant service provider records. There is no better alternative; medical records are reasonably valid for conditions requiring medical diagnosis and monitoring (e.g., diabetes, hypertension) but are less valid for conditions that have important effects on individuals but do not require continued supervision, i.e., chronic knee pain (36).

Assessment of the amount of informal care individuals receive is problematic but important (37). Participants found it difficult to estimate informal care in terms of time. Frequently what we defined as informal care (housework, shopping, gardening, emotional support) were activities that partners, family, and friends normally performed as part of a relationship that participants played down. Some may have been reluctant to admit needing and accepting help. Many coped by adapting their environment and activities (showering rather than bathing) with minimal help. Many ignored nonessential tasks (e.g., decorating) and refrained from or reduced participation in social activities. This might underestimate the amount of informal care needed.

Although the CSRI asked about impacts related to knee pain, this is difficult to disentangle from the impact of other comorbidities common in elderly persons. Consequently, the impact of knee pain may have been overstated inadvertently. Conversely, because knee pain is a major determinant of functional limitation and functional limitation is a risk factor for chronic ill health, the indirect health care costs for chronic conditions due to functional limitations caused by knee pain may be underestimated.

Because most participants were retired, lost productivity and social welfare benefits were small. Although all the participants reported chronic knee pain, health care costs were low, mostly repeat medication. A large proportion of individuals were taking over-the-counter remedies (e.g., fish oil, glucosamine), but few were receiving therapies often sought for chronic pain (e.g., acupuncture, private therapies). Individuals accepted and coped with functioning limitations by decreasing or altering their activities. This enabled them to maximize their independence and minimize the burden on others. Assistance was most frequently necessary for essential arduous tasks (e.g., housework, shopping).

Overall, health professionals provide, and people seem to expect and accept, a low level of care for chronic knee pain. Chronic knee pain is regarded as a benign, inevitable consequence of aging, for which little can be done beyond prolonged medication, regardless of its costs (17) and risks (9,14–16). However, because of the prevalence of knee pain, health and social care expenditure is very great (3,7–9) and likely to escalate as the working life extends (increasing lost productivity, earnings, and social security payments), the population ages, and consultations for musculoskeletal disorders increase (11).

Usual primary care was the cheapest option but least cost-effective. Supplementing this with rehabilitation incurred extra costs, but improved functioning. Group rehabilitation achieved comparable improvement in functioning at half the cost of individual rehabilitation, and therefore was most cost-effective. This finding could be used to justify withdrawing provision of individual therapy. However, because individual sessions can be arranged at convenient times and missed sessions can be re-arranged, removing this flexible management option might deny some people (the employed) effective treatment. It may be preferable to retain this management option but reduce its costs and maximize efficiency by finding the optimal number of individual sessions without compromising effectiveness.

We found little difference in QALYs between the arms. ESCAPE-knee pain was a brief intervention designed to improve functioning and be deliverable to many individuals. It was not expected or designed to enable individuals to function normally, completely alleviate pain, or substantially affect all factors that contribute to quality of life in an elderly, inner-city population (e.g., comorbidity, family and social issues, anxiety, depression, socioeconomic situation). Therefore, quality of life will remain compromised. Moreover, the EQ-5D may be insensitive to quality-of-life change in this patient group (38,39) and the trial was not powered to detect meaningful change in this outcome.

Although intervention costs could be reduced by using junior therapists and/or larger groups, safety issues might necessitate additional personal support, which could negate potential savings. We considered the health and social care perspective to be the most appropriate to health commissioners considering productivity, because lost earnings, sickness benefit, and travel time are not major issues in this retired patient population. Including these issues in a societal perspective increased the costs in all the trial arms, but the broad inferences from the study remain unaltered.

This is the first study integrating exercise and self-management for persons with chronic knee pain. There is evidence that self-management is cost-effective for chronic knee pain (40,41). There is less evaluation of the costs of exercise, but recent studies of similar patient populations demonstrated that prolonged exercise regimens resulted in benefits immediately following completion (42,43) and were cost-effective (44–46). The important differences in this study are that the improved functioning attained during ESCAPE-knee pain was sustained for 6 months, the number needed to treat was smaller (better) (43,45), and the intervention was less costly (44,45).

Interventions for chronic pain conditions will benefit few individuals if they require participation in complex, prolonged interventions that are impractical and too expensive to implement widely. As more individuals live and work longer with chronic ill health, safe, affordable interventions will be needed to maximize functioning, independence, and efficient use of health and societal resources. ESCAPE-knee pain was designed to be a brief, affordable, and cost-effective intervention to improve functioning for many people. We have previously shown it to be safe and effective (24,25). This economic evaluation demonstrates that ESCAPE-knee pain is cost-effective if value is placed on meaningful improvements in functioning.

## AUTHOR CONTRIBUTIONS

Dr. Hurley had full access to all of the data in the study and takes responsibility for the integrity of the data and the accuracy of the data analysis.

**Study design.** Hurley, Walsh, Pimm, Jones, Reeves, Dieppe, Patel.

**Acquisition of data.** Hurley, Walsh, Mitchell.

**Analysis and interpretation of data.** Hurley, Walsh, Pimm, Williamson, Jones, Reeves, Dieppe, Patel.

**Manuscript preparation.** Hurley, Walsh, Mitchell, Pimm, Williamson, Jones, Reeves, Dieppe, Patel.

**Statistical analysis.** Hurley, Williamson, Patel.

**Trial Steering Group members.** Hurley, Walsh, Mitchell, Pimm, Williamson, Jones, Reeves, Dieppe, Patel.
