# Supplementary material for: Economic evaluation of a rehabilitation program integrating exercise, self-management, and active coping strategies for chronic knee pain
Source: Arthritis Rheum. 2007 Oct 15;57(7):1220–9. doi: 10.1002/art.23011 (PMC2675012; doi:10.1002/art.23011)
Supplement: Supplementary file 1 [file art0057-1220-SD1.pdf]

APPENDIX A: UNIT COSTS

Item	Unit cost (£, 2003/2004 prices)	Source	Assumptions
**Inpatient services per inpatient day**			
General medical	287.10	Netten A, Curtis L. Unit costs of health and social care, 2002. PSSRU, University of Kent.	Generic hospital cost assumed. 2001/2002 unit cost of £273.00 inflated using HCHS Pay and Prices Index.
Rehabilitation	201.92	Netten A, Curtis L. Unit costs of health and social care, 2002. PSSRU, University of Kent.	2001/2002 unit cost of £192.00 inflated using HCHS Pay and Prices Index.
Geriatric	152.49	Netten A, Curtis L. Unit costs of health and social care, 2002. PSSRU, University of Kent.	2001/2002 unit cost of £145.00 inflated using HCHS Pay and Prices Index.
General surgery	387.01	Netten A, Curtis L. Unit costs of health and social care, 2002. PSSRU, University of Kent.	2001/2002 unit cost of £368.00 inflated using HCHS Pay and Prices Index.
Rheumatology	253.45	Netten A, Curtis L. Unit costs of health and social care, 2002. PSSRU, University of Kent.	2001/2002 unit cost of £241.00 inflated using HCHS Pay and Prices Index.
Orthopedic	287.10	Netten A, Curtis L. Unit costs of health and social care, 2002. PSSRU, University of Kent.	Generic hospital cost assumed in the absence of data for orthopedic specialty. 2001/2002 unit cost of £273.00 inflated using HCHS Pay and Prices Index.
**Outpatient services per attendance**			
General medical	96.73	Department of Health: National Schedule of Reference Costs, 2003. URL: http://dh.gov.uk/assetRoot/04/07/01/10/04070110.xls. [Accessed 24 January 2005]	Assumed cost of followup attendances, rather than the higher cost of first attendances. 2002/2003 unit cost of £94.00 inflated using the HCHS Pay and Prices Index.
Physiotherapy	49.39	Department of Health: National Schedule of Reference Costs, 2003. URL: http://dh.gov.uk/assetRoot/04/07/01/10/04070110.xls. [Accessed 19 May 2005]	Assumed cost of treatment rather than assessment. 2002/2003 unit cost of £48 inflated using the HCHS Pay and Prices Index.
Geriatric	125.54	Department of Health: National Schedule of Reference Costs, 2003. URL: http://dh.gov.uk/assetRoot/04/07/01/10/04070110.xls. [Accessed 24 January 2005]	Assumed cost of followup attendances, rather than the higher cost of first attendances. 2002/2003 unit cost of £122.00 inflated using the HCHS Pay and Prices Index.
General surgery	77.18	Department of Health: National Schedule of Reference Costs, 2003. URL: http://dh.gov.uk/assetRoot/04/07/01/10/04070110.xls. [Accessed 24 January 2005]	Assumed cost of followup attendances, rather than the higher cost of first attendances. 2002/2003 unit cost of £75.00 inflated using the HCHS Pay and Prices Index.
Rheumatology	81.30	Department of Health: National Schedule of Reference Costs, 2003. URL: http://dh.gov.uk/assetRoot/04/07/01/10/04070110.xls. [Accessed 24 January 2005]	Assumed cost of followup attendances, rather than the higher cost of first attendances. 2002/2003 unit cost of £79.00 inflated using the HCHS Pay and Prices Index.
Orthopedic	63.80	Department of Health: National Schedule of Reference Costs, 2003. URL: http://dh.gov.uk/assetRoot/04/07/01/10/04070110.xls.	Assumed cost of followup attendances, rather than the higher cost of first attendances. 2002/2003 unit cost of £62.00 inflated using the HCHS Pay and Prices Index.

		[Accessed 24 January 2005]	
Pain clinic	77.18	Department of Health: National Schedule of Reference Costs, 2003. URL: http://dh.gov.uk/assetRoot/04/07/01/10/04070110.xls. [Accessed 24 January 2005]	Assumed cost of followup attendances, rather than the higher cost of first attendances. 2002/2003 unit cost of £75.00 inflated using the HCHS Pay and Prices Index.
Endoscopy	200.18	Department of Health: National Schedule of Reference Costs, 2003. URL: http://dh.gov.uk/assetRoot/04/07/01/10/04070110.xls. [Accessed 24 January 2005]	Assumed cost of diagnostic endoscopy test. 2002/2003 unit cost of £194.53 inflated using the HCHS Pay and Prices Index.
Hydrotherapy	33.33	Epps H, Ginnelly L, Utley M, Southwood T, Gallivan S, Sculpher M, et al. Is hydrotherapy cost-effective? The costs and outcomes of hydrotherapy programmes compared with physiotherapy land techniques in children with rheumatic conditions. Health Technol Assess. In Press.<br>Curtis L, Netten A. Unit costs of health and social care, 2004. PSSRU, University of Kent.	Unit cost derived from data in Epps et al. Fixed costs were reported as £17.19 per session at 2000 prices, based on top-down calculations on data supplied by Great Ormond Street Hospital, Alderhey Hospital, and University College London Hospital. Inflating this to 2003/2004 prices using the HCHS Pay and Prices Index gives a fixed cost of £18.60. Variable costs were derived based on the reported average session length of 29 minutes. Assuming that the session is provided by one hospital-based physiotherapist, the salary cost of 29 minutes of client contact is estimated to be £14.73, based on figures in Curtis and Netten, 2004. Therefore, the total cost per session is estimated at £33.33.
Acupuncture	54.20	Lindall S. Is acupuncture for pain relief in general practice cost-effective? Acupunct Med 1999;17:97---100.	Based on charge to the general practice fund by the acupuncturist of £46.00 per attendance, at 1998/1999 prices. Inflated to 2003/2004 prices using the HCHS Pay and Prices Index.
Accident and emergency	57.63	Department of Health: National Schedule of Reference Costs, 2003. URL: http://dh.gov.uk/assetRoot/04/07/01/10/04070110.xls. [Accessed 24 January 2005]	Assumed cost of followup attendances, rather than the higher cost of first attendances. 2002/2003 unit cost of £56.00 inflated using the HCHS Pay and Prices Index.
**Other hospital services per visit**			
Radiograph	30.64	Bryan S, Bungay HP, Weatherburn G, Field S. Magnetic resonance imaging for investigation of the knee joint: a clinical and economic evaluation. Int J Technol Assess Health Care 2004;20:222---9.	Cost of knee radiograph, supplied by University Hospital Birmingham. 1998 unit cost of £25.00 inflated using HCHS Pay and Prices Index.
MRI scan	272.69	Department of Health: National Schedule of Reference Costs, 2003. URL: http://dh.gov.uk/assetRoot/04/07/01/10/04070110.xls. [Accessed 19 May 2005]	2002/2003 unit cost of rheumatology MRI scan of £265.00 inflated using the HCHS Pay and Prices Index.
Ultrasound scan	92.61	Department of Health: National Schedule of Reference Costs, 2003. URL: http://dh.gov.uk/assetRoot/04/07/01/10/04070110.xls. [Accessed 19 May 2005]	2002/2003 unit cost of rheumatology ultrasound scan of £90.00 inflated using the HCHS Pay and Prices Index.

Arthroscopy	594.35	Bryan S, Bungay HP, Weatherburn G, Field S. Magnetic resonance imaging for investigation of the knee joint: a clinical and economic evaluation. Int J Technol Assess Health Care 2004;20:222---9.	Cost of arthroscopy procedure from survey of 10 NHS Trusts. 1998 unit cost of £485.00 inflated using HCHS Pay and Prices Index.
Blood test	3.42	Department of Health: National Schedule of Reference Costs, 2003. URL: http://dh.gov.uk/assetRoot/04/07/01/10/04070110.xls. [Accessed 19 May 2005]	2002/2003 average unit cost for hematology tests of £3.32 inflated using the HCHS Pay and Prices Index.
Orthotist	96.73	Department of Health: National Schedule of Reference Costs, 2003. URL: http://dh.gov.uk/assetRoot/04/07/01/10/04070110.xls. [Accessed 24 January 2005]	Assumed cost of general medical outpatient visit in absence of relevant unit cost. Assumed cost of followup attendances, rather than the higher cost of first attendances. 2002/2003 unit cost of £94.00 inflated using the HCHS Pay and Prices Index.
Day hospital	90.88	Netten A, Curtis L. Unit costs of health and social care, 2002. PSSRU, University of Kent.	Cost of generic day hospital attendance. 2002/2003 unit cost of £86.00 inflated using the HCHS Pay and Prices Index.
**Community-based services per relevant unit of contact**			
GP at the surgery, per minute	1.72	Curtis L, Netten A. Unit costs of health and social care, 2004. PSSRU, University of Kent.	Excludes direct care staff and qualification costs.
GP at the surgery, per contact	16.00	Curtis L, Netten A. Unit costs of health and social care, 2004. PSSRU, University of Kent.	Excludes direct care staff and qualification costs.
GP home visit, per minute	2.69 + 5.00 travel cost	Curtis L, Netten A. Unit costs of health and social care, 2004. PSSRU, University of Kent.	Excluding direct care staff and qualification costs. Includes cost of travel time.
GP home visit, per visit	50.00	Curtis L, Netten A. Unit costs of health and social care, 2004. PSSRU, University of Kent.	Excludes direct care staff and qualification costs. Includes cost of travel time and travel cost.
Practice nurse at the surgery, per minute	0.41	Curtis L, Netten A. Unit costs of health and social care, 2004. PSSRU, University of Kent.	Excludes qualification costs.
District nurse at clinic, per minute	0.60	Curtis L, Netten A. Unit costs of health and social care, 2004. PSSRU, University of Kent.	Excludes qualification costs.
District nurse at clinic, per visit	12.00	Curtis L, Netten A. Unit costs of health and social care, 2004. PSSRU, University of Kent.	Excludes qualification costs. As average clinic contact time was unavailable, same contact duration as for home visits was used (20 minutes).
District nurse home visit, per minute	0.83 + 1.20 travel cost	Curtis L, Netten A. Unit costs of health and social care, 2004. PSSRU, University of Kent.	Excludes qualification costs.
District nurse home visit, per visit	18.00 + 1.20 travel cost	Curtis L, Netten A. Unit costs of health and social care, 2004. PSSRU, University of Kent.	Excludes qualification costs.
Physiotherapist at surgery, per minute	0.67	Curtis L, Netten A. Unit costs of health and social care, 2004. PSSRU, University of Kent.	Excludes qualification costs.
Physiotherapist home visit, per minute	0.68 + 2.30 travel cost	Curtis L, Netten A. Unit costs of health and social care, 2004. PSSRU, University of Kent.	Excludes qualification costs.
Occupational therapist at surgery, per minute	0.67	Curtis L, Netten A. Unit costs of health and social care, 2004.	Excludes qualification costs.

		PSSRU, University of Kent.	
Social worker home visit, per minute	1.65	Curtis L, Netten A. Unit costs of health and social care, 2004. PSSRU, University of Kent.	
Acupuncture general practice visit, per visit	54.20	Lindall S. Is acupuncture for pain relief in general practice cost-effective? Acupunct Med 1999;17:97---100.	Based on charge to the general practice fund by the acupuncturist of £46.00 per attendance, at 1998/1999 prices. Inflated to 2003/2004 prices using the HCHS Pay and Prices Index.
Homeopathy private clinic visit, per visit	32.25	Wonderling D, Vickers AJ, Grieve R, McCarney R. Cost effectiveness analysis of a randomised trial of acupuncture for chronic headache in primary care. BMJ 2004;328:747.	Based on patient-reported data in a sample of 401 patients recruited from general practices in England and Wales. 2002/2003 cost (£31.46) inflated to 2003/2004 prices using the HCHS Prices Index.
Osteopathy clinic visit, per visit	37.50	General Osteopathic Council. URL: http://www.osteopathy.org.uk/about_osteo/Arthritis.pdf. [Accessed 2 February 2005]	Based on average of reported range of £25.00 to £50.00 for a 30---40-minute session.
**Medications**			
At baseline, for a 6 month supply	Range 1.92---136.81	Department of Health. Prescription cost analysis: England, 2004. Department of Health, London. URL: http://www.dh.gov.uk/assetRoot/04/10/76/26/04107626.pdf. [Accessed 10 May 2005]. Joint Formulary Committee (September 2004). British National Formulary. 48th ed. London: British Medical Association and Royal Pharmaceutical Society of Great Britain.	
At 6 weeks, for a 1.38-month supply	Range 0.44---31.57	Department of Health. Prescription cost analysis: England, 2004. Department of Health, London. URL: http://www.dh.gov.uk/assetRoot/04/10/76/26/04107626.pdf. [Accessed 10 May 2005] Joint Formulary Committee (September 2004). British National Formulary. 48th ed. London: British Medical Association and Royal Pharmaceutical Society of Great Britain.	
At 6 months, for a 4.62-month supply	Range 1.47---105.23	Department of Health. Prescription cost analysis: England, 2004. Department of Health, London. URL: http://www.dh.gov.uk/assetRoot/04/10/76/26/04107626.pdf. [Accessed 10 May 2005] Joint Formulary Committee (September 2004). British National Formulary. 48th ed. London: British Medical	

		Association and Royal Pharmaceutical Society of Great Britain.	
**Social security benefits**			
Income support	57.22	Department for Work and Pensions (2004). Benefits and services. URL: http://www.dwp.gov.uk/lifeevent/benefits/index.asp#I. [Accessed 28 June 2004]	October 2002 weekly rate of 55.65 inflated using the Retail Price Index. Personal allowances for single people age ≥25.
Housing benefit	75.93	Department for Work and Pensions. Benefits and services. URL: http://www.dwp.gov.uk/lifeevent/benefits/index.htm. [Accessed 28 June 2004]	2002 weekly rates of 73.85 inflated using the Retail Price Index. Age ≥18 and receiving income support. Includes personal allowance for single people age ≥25.
Statutory sick pay	64.34	Department for Work and Pensions (2004). Benefits and services. URL: http://www.dwp.gov.uk/lifeevent/benefits/index.asp#I. [Accessed 28 June 2004]	April 2004 weekly rate of 66.15 deflated using the Retail Price Index. Standard rate for those with average gross weekly earnings of £79.00.
Disability living allowance	33.13	Department for Work and Pensions (2004). Benefits and services. URL: http://www.dwp.gov.uk/lifeevent/benefits/index.asp#. [Accessed 28 June 2004]	April 2004 weekly rate of 34.06 deflated using the Retail Price Index. Average of 5 rates available for those who need looking after and those who need help with mobility.
Incapacity benefit	70.66	Department for Work and Pensions (2004). Benefits and services. URL: http://www.dwp.gov.uk/lifeevent/benefits/index.asp#I. [Accessed 28 June 2004]	April 2004 weekly rate of 72.65 deflated using the Retail Price Index. Average of higher and lower rate available for those over state pension age.
Attendance allowance	47.74	Department for Work and Pensions (2004). Benefits and services. URL: http://www.dwp.gov.uk/lifeevent/benefits/index.asp#I. [Accessed 28 June 2004]	April 2004 weekly rate of 49.08 deflated the Retail Price Index. Average of higher and lower rate.
State pension	40.84	Department for Work and Pensions. Benefits and services. URL: http://www.dwp.gov.uk/lifeevent/benefits/index.htm. [Accessed 28 June 2004]	2002 weekly rates of 39.72 inflated using the Retail Price Index. Mean rate for basic pension.
Occupational pension	127.00	Department for Work and Pensions (2004). The pensioners' income series 2002/2003. URL: http://www.dwp.gov.uk/asd/asd6/PI_series_0203.pdf. [Accessed 18 May 05]	Average amount of occupational pension income for those in receipt, per week at 2002/2003 prices. 2003/2003 rate of £122 inflated using the Gross Domestic Product Inflator.
**Lost productivity**			
National average wage per hour	12.07	National Statistics. New Earnings Survey 2002. URL: http://www.statistics.gov.uk/downloads/theme_labour/NES2002_GB/NES2002_Streamlined_analyses.pdf.	Average gross hourly earnings, excluding overtime, for full-time employees on adult rates whose pay was not affected by absence, in all industries and services in Great Britain in April 2002. 2002 rate of 11.73 inflated using the Gross Domestic Product Inflator.

		[Accessed 28 June 2004]
**Informal care**		
UK minimum wage per hour	4.50	Department of Trade and Industry. National minimum wage annual report, 2002/2003. URL: http://www.dti.gov.uk/er/nmw/ar2003.pdf. [Accessed 25 January 2005]

* HCHS = Hospital and community health services; MRI = magnetic resonance imaging; NHS = National Health Service; GP = general practitioner.
